# Say hello to my little friend… micronutraceuticals in neuroenergetics, neuronal health, and neurodegenerative diseases

**DOI:** 10.3389/fnins.2025.1498655

**Published:** 2025-04-23

**Authors:** Shayne Mason

**Affiliations:** Human Metabolomics, North-West University, Potchefstroom, South Africa

**Keywords:** vitamins, neuroenergetics, brain, energy metabolism, neurons, neurodegenerative disease

## Abstract

Vitamins and minerals (micronutraceuticals) maintain good health. However, the specific effects of these micronutraceuticals on brain health are often overlooked, or not even known. In this review, an overview of the direct and indirect effects of micronutraceuticals on brain energy metabolism (neuroenergetics) and neuronal health is provided. Thereafter, a holistic summary of the existing studies that have shown the impact of micronutraceuticals on neurodegenerative diseases. Lastly, this review concludes by identifying several research gaps that remain and provides suggestions for future research on these hot topics.

## 1 Introduction

The term “nutraceutical,” first coined in 1989, refers to dietary foods with medicinal and health benefits (Andlauer and Fürst, 2002). In the context of this review, the term “micronutraceuticals” will be used to describe all micronutrients that are inexplicably intertwined with nutraceuticals. These micronutraceuticals are necessary for the functionality of nutraceuticals and include vitamins (A, B, C, D, and E) and minerals (Ca, Cu, Fe, Zn, Mg, etc.). Vitamins encompass a vast array of polar (water-soluble) and non-polar (fat-soluble) chemical compounds that are classified as either non-essential (can be synthesized in our bodies) or essential (can only be obtained from exogenous sources—dietary). While the focus of this review is on vitamins, several minerals are also mentioned as cofactors of important metabolic reactions. The significance of minerals in brain health should not be undermined and deserves equivalent attention to vitamins, but perhaps in another review. Of the vitamins discussed here, most fall into the B group because there is a paucity of information on the other vitamins.

We should have a well-balanced diet to obtain all the vitamins that are needed for healthy living. Even if our diet is well balanced, once we reach middle age and progress to the age of the “elderly,” vitamin supplements are strongly recommended to maintain healthy neurological function (O’Leary et al., 2012). There is increasing scientific evidence that vitamin supplementation can reduce cognitive decline associated with advanced aging (Aisen et al., 2008; O’Leary et al., 2012). Moreover, neuropathological diseases often arise because of perturbed brain energy metabolism (neuroenergetics) (Mason, 2017) and/or physiological changes that impact neuronal health. The roles of micronutraceuticals (“the little guys”) in neuroenergetics and neuronal health are often overlooked.

Hence, the aim of this review is to: (1) give an overview of the role of micronutraceuticals in neuroenergetics; (2) describe the physiological association these micronutraceuticals have with maintaining healthy neuronal cells; (3) relate some of the major neurodegenerative diseases to altered micronutraceuticals; and lastly, (4) identify research gaps and directives for future research on this topic.

## 2 Micronutraceuticals in neuroenergetics

Homeostatic neuroenergetics (that is, adequate brain energy metabolism) are closely linked to normal neuronal function and brain health. However, homeostasis of the brain is quickly lost when the metabolic pathways associated with neuroenergetics are perturbed (Mason, 2017; Falkowska et al., 2015). Quite often, allostatic overload and/or pathology caused by altered neuroenergetics can be linked to hypovitaminosis—a deficiency in one or more vitamins, as shown in this review.

**FIGURE 1:**
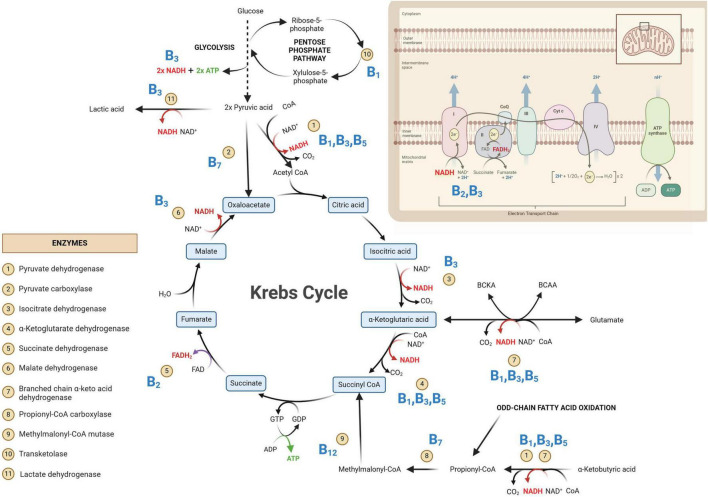
Primary metabolic pathways involved in neuroenergetics [glycolysis, Krebs cycle and oxidative phosphorylation (OXPHOS)]. Some of the key enzymes are indicated and their associated B vitamins (given in bold blue text) are required for homeostasis of neuroenergetics. NAD, oxidized nicotinamide adenine dinucleotide; NADH, oxidized nicotinamide adenine dinucleotide; FAD, oxidized flavin adenine dinucleotide; FADH_2_, reduced flavin adenine dinucleotide; GDP, guanosine diphosphate; GTP, guanosine triphosphate; ADP, adenine diphosphate; ATP, adenine triphosphate; CoA, coenzyme A; CO_2_, carbon dioxide.

The brain is the highest energy-consuming organ in the human body, consuming 25% of circulating glucose under normal conditions (Pellerin, 2010). In the brain, primary energy metabolism is predominantly glucocentric—relying mostly on glucose, albeit with shifting paradigms (Mason, 2017). The catabolism of glucose through primary energy metabolic pathways involved in neuroenergetics [glycolysis, Krebs cycle, and oxidative phosphorylation (OXPHOS)] is illustrated in Figure 1. In the case of inborn errors of metabolism (IEMs), any genetic disorder that results in a defective enzyme involved in any of these energy metabolic pathways can lead to serious consequences, often death at an early age, if left untreated. It is also important to note that many enzymes involved in neuroenergetics are dependent on coenzymes and/or cofactors (micronutraceuticals) for their normal functions. Hence, hypovitaminosis can result in metabolic conditions that subtly mimic secondary forms of IEMs.

In Figure 1, the glycolysis metabolic pathway is condensed to that of the primary substrate glucose and the end-product pyruvate. The net yield of glycolysis from one glucose molecule is two units of adenosine triphosphate (ATP; the energy currency of the cells) and two units of reduced nicotinamide adenine dinucleotide (NADH). The emphasis in Figure 1 is on the Krebs cycle—the “heart” of metabolism, which will be discussed in greater detail. The OXPHOS system is also depicted in Figure 1, indicating the entry points of the all-important reduced coenzymes [NADH and reduced flavin adenine dinucleotide (FADH_2_)] into the electron transport chain. Of note within Figure 1 are the eleven indicated enzymes and vitamins (shown in bold blue text in Figure 1) associated with these enzymes. What immediately stands out in Figure 1 is that all these vitamins are B-group vitamins. Another point of interest in Figure 1 is that seven of the eleven listed enzymes are dehydrogenases.

**FIGURE 2:**
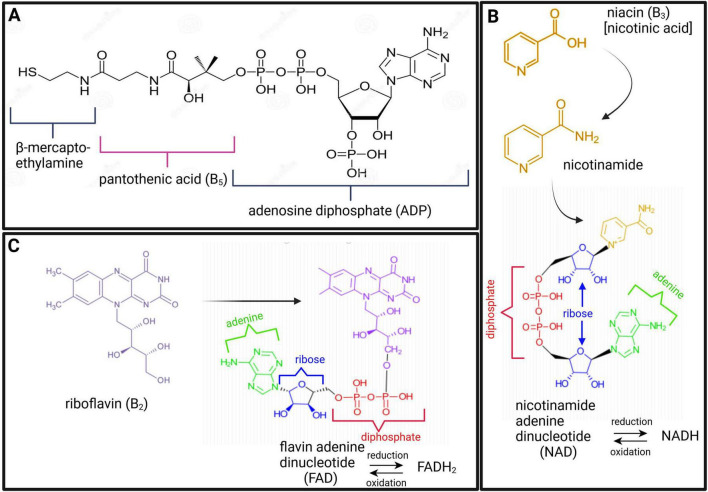
Chemical structures of coenzyme A [CoA; **(A)**], nicotinamide adenine dinucleotide [NAD; **(B)**], and flavin adenine dinucleotide [FAD; **(C)**], highlighting the constituents of vitamins B_5_ (maroon), B_3_ (gold), and B_2_ (purple), respectively.

Dehydrogenases are oxidoreductase enzymes that catalyze either oxidative or reductive metabolic reactions, depending on the direction of the metabolic reaction. Each dehydrogenase enzyme requires a coenzyme that facilitates the transfer of electrons and hydrogen atoms (H^+^). The coenzymes that function with dehydrogenase are typically NAD^+^→ NADH + H^+^ and, to a lesser extent, FAD^+^→ FADH_2_ + H^+^. If we look at the chemical structure of these coenzymes (Figure 2), it starts to become clear why vitamins (in particular B_2_ and B_3_) are important. In Figure 2, it is shown that vitamin B_3_ (niacin/nicotinic acid) is converted to its amide form nicotinamide, which is then incorporated into the structure of NAD. Similarly, for FAD, vitamin B_2_ (riboflavin) forms part of the chemical structure of FAD (shown in Figure 2). Hence, deficiencies in vitamins B_2_ and B_3_ can lead to insufficient levels of the coenzymes FAD and NAD, respectively. Insufficient NAD and FAD will result in decreased production of their reduced forms (NADH and FADH_2_) and a noticeable decrease in OXPHOS, leading to impaired neuroenergetics. Additionally, increased NAD/NADH and FAD/FADH_2_ ratios are biochemical indicators of oxidative stress, which will be discussed in the following sections. Another important coenzyme that is needed for normal neuroenergetics is coenzyme A (CoA). The metabolic reactions of pyruvic acid → acetyl-CoA, α-ketoglutaric acid → succinyl-CoA, and α-ketobutyric acid → propionyl-CoA require the presence of CoA. Figure 2 shows the chemical structure of CoA, and it is clearly indicated that the core component of this coenzyme is vitamin B_5_ (pantothenic acid). Hence, vitamin B_5_ deficiency will result in CoA deficiency and a subsequent perturbation in neuroenergetics. Lastly, vitamin B_1_ (thiamine) is also required for the proper functioning of dehydrogenases. Pyruvate dehydrogenase (Krebs cycle), α-ketoglutarate dehydrogenase (Krebs cycle), and branched chain α-keto acid dehydrogenase (involvement of glutamate and branched-chain amino acids in Krebs cycle), as well as transketolase (pentose phosphate pathway), are enzymes that are thiamine-dependent and are important for neuroenergetics (see Figure 1). Because these four enzymatic reactions involve the creation of reducing power (NADH), thiamine thus fights oxidative stress (i.e., it has anti-oxidative properties), making thiamine neuroprotective (discussed in more detail in the next section). Downregulation of pyruvate dehydrogenase and α-ketoglutarate dehydrogenase leads to an interruption of the Krebs cycle, resulting in reduced ATP production in the brain (i.e., reduced neuroenergetics). This loss of ATP also results in calcium overflow in the brain, leading to neuronal apoptosis. It should also be noted that another important mineral in neuroenergetics is magnesium because ATP must bind to magnesium for it to be biologically active. Hence, magnesium deficiency has a global effect on neuroenergetics.

Two of the eleven enzymes in Figure 1 are carboxylases (pyruvate carboxylase and propionyl-CoA carboxylase). Carboxylases catalyze decarboxylation reactions—removal of carboxyl groups and release of carbon dioxide. These carboxylases are dependent on vitamin B_7_ (biotin). Hence, vitamin B_7_ deficiency leads to reduced activity of pyruvate carboxylase (pyruvate → oxaloacetate) and propionyl-CoA carboxylase (propionyl-CoA → methylmalonyl-CoA). Both of these metabolic reactions are also part of the primary energy pathway (Figure 1); hence, a biotin deficiency can result in perturbed neuroenergetics. Lastly, vitamin B_12_ (cobalamine) is required for the proper functioning of methylmalonyl-CoA mutase, for propionyl-CoA, which is produced by odd-chain fatty acid oxidation, to be incorporated into the Krebs cycle. It should be noted that the other end-product of fatty acid oxidation – acetyl-CoA, is also incorporated into the Krebs cycle for energy production, reinforcing the statement that the Krebs cycle is the “heart” of neuroenergetics.

Thus, as described above and illustrated in Figures 1, 2, vitamins B_1_, B_2_, B_3_, B_5_, B_7_, and B_12_, as well as magnesium, are all necessary micronutraceuticals for the homeostasis of neuroenergetics. A deficiency in one or a combination of these micronutraceuticals will result in allostatic overload. If this allostatic overload is not alleviated (i.e., if this micronutraceutical deficiency is not corrected), the health of the neurons will begin to deteriorate, causing neurodegeneration.

## 3 Micronutraceuticals and neuronal health

Perturbed neuroenergetics are sufficient to cause neuronal dysfunction, or even neuronal death; however, beyond neuroenergetics, micronutraceuticals have direct involvement in various biological functions that affect the health of neuronal cells (summarized in Table 1), as discussed here.

**TABLE 1:** Summary of the various roles that micronutraceuticals play in neuronal health.

Micronutraceutical	Role in neuronal health	References
Thiamine (B_1_)	Neuro-modulatory	Beltramo et al., 2008; Hirsch and Parrott, 2012
	Formation of synapses, axonal growth, and myelinogenesis	Bâ, 2005
	Nerve stimulation, structure and function of neuronal membranes, and nerve membrane functions (i.e., regulates ion channels – Na + gating and activating chloride ion channels)	Bâ, 2008; Bender, 1999; Spector and Johanson, 2007
	Myelin maintenance (contributes to nerve conduction velocity)	Martin, 2001
	Neuroprotective effects against excess glutamate	Geng et al., 1995
	Neurotransmitters: acetylcholine, dopamine, gamma-aminobutyric acid, and noradrenaline	Beltramo et al., 2008; Hirsch and Parrott, 2012; Martin, 2001; Moretti and Peinkhofer, 2019
Riboflavin (B_2_)	Anti-oxidative – glutathione redox cycle	Ashoori and Saedisomeolia, 2014; Moretti and Peinkhofer, 2019; Plantone et al., 2021
	Neuroprotective – direct inhibition of glutamate neuronal release, and anti-inflammatory	Moretti and Peinkhofer, 2019
	Protects against neurotoxicity by ameliorating oxidative stress, mitochondrial dysfunction, and neuroinflammation	Marashly and Bohlega, 2017
Niacin/nicotinamide (B_3_)	Anti-inflammatory (modulates neuroinflammation by regulating microglia)	Moretti and Peinkhofer, 2019; Wakade and Chong, 2014
	Direct neurotransmitter and increased dopamine synthesis	Moretti and Peinkhofer, 2019; Wakade and Chong, 2014
	Anti-oxidative and promotes calcium signaling	Moretti and Peinkhofer, 2019
	Key mediator in neuronal survival and development	Gasperi et al., 2019
	Neuroprotective (protects neurons from axonal degeneration)	Vaur et al., 2017; Wakade and Chong, 2014
Pantothenic acid (B_5_)	Required for the synthesis of coenzyme A (important for neuroenergetics, acetylation of metabolites and metabolism of lipids and steroids), neurotransmitters, and steroid hormones	Kennedy, 2016; Moretti and Peinkhofer, 2019; Rucker and Bauerly, 2013
	Myelin structure and function	Ismail et al., 2020
	Core component of acyl carrier proteins (ACPs) used in fatty acid metabolism (important for neuronal health)	Rucker and Bauerly, 2013
	Anti-oxidative	Moretti and Peinkhofer, 2019
Pyridoxine (B_6_)	Affects adrenergic, glutamergic, and serotonergic systems	Calderón-Ospina and Nava-Mesa, 2020
	Sphingolipid (myelin) synthesis	Selhub et al., 2000; Spinneker et al., 2007
	Anti-oxidative (glutathione metabolism)	Wendołowicz et al., 2018
	Neuroprotective [regulates glutamate (excitotoxity) levels]	Calderón-Ospina and Nava-Mesa, 2020; Dakshinamurti et al., 2003
	Phospholipid metabolism	Dakshinamurti and Dakshinamurti, 2013
	Protects cerebral endothial cells against oxidative damage and prevents blood-brain barrier dysfunction	Moretti and Peinkhofer, 2019
	Neurotransmitters: dopamine, norepinephrine, serotonin, and gamma-aminobutyric acid	Calderón-Ospina and Nava-Mesa, 2020; Dakshinamurti and Dakshinamurti, 2013
Biotin (B_7_)	Glucose homeostasis	Kennedy, 2016
	Anti-oxidative and neuroprotective	Moretti and Peinkhofer, 2019
Cobalamine (B_12_)	Anti-oxidative (glutathione metabolism)	Wendołowicz et al., 2018; Chan et al., 2018; Manzanares and Hardy, 2010; Moreira et al., 2011
	Growth, differentiation, development, and repair of neurons	Reynolds, 2006
	Promotes glucose uptake in the brain and stimulates neuronal survival	Zhou et al., 2023
Ascorbic acid (C)	Anti-oxidative	García-Krauss et al., 2016; Harrison et al., 2010; Narne et al., 2017
	Regenerates vitamin E to protect neurons against lipid peroxidation	Li et al., 2003
	Optimal neuronal functioning (neurotransmission, synaptic maturation and neuronal differentiation), regulates cellular response during hypoxic conditions, and neuroprotective (glutamate clearance)	Narne et al., 2017
	Oxidized form of vitamin C – dehydroascorbic acid, promotes the death of stressed neuronal cells	García-Krauss et al., 2016
	High abundance in brain regions rich in neurons (e.g., hippocampus)	Harrison et al., 2010
Calcitriol (D), ergocalciferol (D_2_), and cholecalciferol (D_3_)	Regulation of neurotransmitters, neuronal differentiation, axonal growth, voltage-sensitive calcium channels, neurotrophic factors, and ROS	Eyles et al., 2013
	Axon regeneration	Chabas et al., 2008
	Promotes neurite outgrowth	Brown et al., 2003; Neveu et al., 1994
	Neuroprotective effects reduce excitotoxicity (regulates glutamate and modulates NMDA receptors) and ROS-induced neurotoxicity, increases glutathione synthesis, and reduces microglial activation	Garcion et al., 2002; Holton, 2021; Ibi et al., 2001
	Immunomodulatory and involved in the synthesis of neurotransmitters and neurotropic factors	Garcion et al., 2002
	Maintain Ca^2+^ homeostasis	Manzanos et al., 2022
α-Tocopherol (E)	Neuroprotective and anti-oxidative role against ROS	Osakada et al., 2003; Tomé et al., 2010
	Anti-inflammatory	Betti et al., 2011
Magnesium	Protects the N-methyl-D-aspartate (NMDA) receptor against uncontrolled calcium ion influx	Domitrz and Cegielska, 2022
	Neuroprotective (regulates glutamate excitotoxicity)	Domitrz and Cegielska, 2022; Murata et al., 2016

Firstly, many micronutraceuticals have anti-oxidative effects. One way in which these micronutraceuticals function as anti-oxidants is that they directly or indirectly neutralize free radicals and excitotoxic compounds in the brain. A good example is the excitotoxic metabolite glutamate, which is released into the synapses of neurons when presynaptic neurons polarize and can initiate the action potential of postsynaptic neurons (Hübel et al., 2017). Vitamins B_1_, B_6_, C, and E, as well as magnesium, are directly involved in glutamate clearance and have a neuroprotective role (see Table 1). Another way that micronutraceuticals are anti-oxidative agents is that they help maintain the redox cycle of other anti-oxidants, such as the glutathione cycle (see Table 1—vitamins B_2_, B_6_, B_12_, and D). Hence, micronutraceuticals regulate oxidants in the brain, protecting neurons.

Secondly, the anti-inflammatory effects of micronutraceuticals further highlight their neuroprotective effects, which go hand-in-hand with their anti-oxidant abilities. Vitamins B_2_, B_3_, C, D, and E (Table 1) have been shown to exhibit anti-inflammatory roles by mitigating oxidative stress and regulating microglial activation (modulating the release of cytokines/chemokines). Additional neuroprotective roles of micronutraceuticals arise through the maintenance of calcium homeostasis (by vitamin D), protection against uncontrolled calcium ion influx (by magnesium), and regeneration of neuroprotective vitamin E (by vitamin C). Indeed, micronutraceuticals represent a unique weapon against the “neurotoxic triad” of excitotoxicity, oxidative stress, and neuroinflammation (Holton, 2021).

Thirdly, the physical structure of neurons (biogenesis, growth, and maintenance) is also dependent on micronutraceuticals. Myelin—the sheath surrounding the axons of neurons—requires vitamins B_1_, B_5_, and B_6_ (see Table 1) for its synthesis and maintenance. Vitamins B_1_ and C are involved in axonal growth and regeneration (see Table 1). Iron is necessary for neuron dendrite growth and branching (Bastian et al., 2016; Brunette et al., 2010). Vitamin B_12_ plays an all-around role as it is a major component required for the growth, differentiation, development, and repair of neurons (Reynolds, 2006). Okada et al., 2010) demonstrated that methylcobalamine is the most effective analog of vitamin B_12_ for promoting neurite outgrowth and neuronal survival through the methylation cycle and that methylcobalamine also promotes nerve regeneration and functional recovery in a rat model of sciatic nerve injury. Of note, vitamins B_1_, B_6_, and B_12_ are often termed “neurotropic B vitamins” due to their joint role in neuronal health and repair (Calderón-Ospina and Nava-Mesa, 2020; Paez-Hurtado et al., 2023). Additionally, since vitamins B_9_ and B_12_ are also closely linked with each other, vitamin B_9_ can also be considered neurotropic.

Lastly, micronutraceuticals are required for normal neuronal function. Nerve conduction and stimulation via gated ion channels are supported by the presence of vitamin B_1_ (see Table 1). Vitamins B_1_ and D also have neuro-modulatory and immune-modulatory functions, respectively. Furthermore, neurons function through neurotransmitters, and micronutraceuticals have been linked to the synthesis and functioning of specific neurotransmitters (e.g., acetylcholine, dopamine, gamma-aminobutyric acid, noradrenaline, and serotonin—see Table 1). For example, vitamin B_6_ is needed for the functioning of aromatic L-amino acid decarboxylase, an enzyme required for the decarboxylation of L-3,4-dihydroxyphenylalanine (L-DOPA) to dopamine and 5-hydroxytryptophan (5-HTP) to serotonin. Hence, vitamin B_6_ is important for normal neuronal function. Vitamins B_6_, B_9_, and B_12_ are typically discussed together as their complementary roles are inextricably linked (Kennedy, 2016). One very good example of this is the homocysteine-methionine cycle and one-carbon metabolism. For more information, the reader is referred to the following papers that cover this specific topic in detail: Araújo et al., 2015; Calderón-Ospina and Nava-Mesa, 2020; Kennedy, 2016; Lauer et al., 2022; Mitchell et al., 2014; Obeid et al., 2007; Reynolds, 2006; Sechi et al., 2016; Smith, 2008; Vogel et al., 2009 Zhang et al., 2009.

## 4 Neuropathologies linked to micronutraceutical deficiencies

It has long been taught at medical schools that deficiencies of certain B vitamins, e.g., B_1_ (beriberi), B_3_ (pellagra) and B_12_, contribute to causes of dementia and other neurological problems. Table 2 presents the results of various studies that identified micronutraceuticals as playing an important role in the pathogenesis and progression of neurodegenerative diseases. Although Table 2 is by no means exhaustive nor comprehensive, it is quite evident that Alzheimer’s disease (AD) is one of the more researched neurodegenerative diseases in terms of micronutraceuticals (Mielech et al., 2020). Missing from Table 2 are psychiatric disorders and other neurological problems, such as headaches, migraines, epilepsy, neuroinfectious diseases, and various rarer genetic diseases that present with major neurological complications. In terms of psychiatric disorders, depression is one the most common psychiatric problems in our society, but some studies have shown benefits by supplementation with vitamins B_1_ (Amerikanou et al., 2023; Rouhani et al., 2023), B_6_ (Almeida et al., 2014), B_9_ (Almeida et al., 2014, 2015; Obeid et al., 2007; Petridou et al., 2016), B_12_ (Almeida et al., 2014, 2015; Moorthy et al., 2012; Obeid et al., 2007; Petridou et al., 2016), and D (Guzek et al., 2023). Bipolar disorder and schizophrenia, two other psychiatric disorders, have been shown to improve with supplementation with vitamin D (Ashton et al., 2021; Gabriel et al., 2023; İmre et al., 2023; Marazziti et al., 2023; Späth et al., 2023) and vitamin B_9_ (Obeid et al., 2007), respectively. Therefore, these studies show that micronutraceuticals should be incorporated into the treatment of psychiatric disorders.

**TABLE 2:** Studies linking deficiencies in micronutraceuticals to major neurodegenerative diseases.

Micronutraceutical	Alzheimer’s disease	Parkinson’s disease	Dementia	Multiple sclerosis	Huntington’s disease	Amyotrophic lateral sclerosis
Thiamine (B_1_)	✓Bender, 1999; Lu’o’ng and Nguy?n, 2011; Kola et al., 2023	✓Kola et al., 2023	✓Gibson et al., 2016; O’Keeffe et al., 1994	✓Nemazannikova et al., 2018	–	–
Riboflavin (B_2_)	–	✓Coimbra and Junqueira, 2003; Marashly and Bohlega, 2017; Murakami et al., 2010; Plantone et al., 2021	✓Plantone et al., 2021; Naghashpour et al., 2017	–	–	–
Niacin (B_3_)	–	✓Wakade and Chong, 2014; Wakade et al., 2015	✓Hegyi et al., 2004; Halubiec et al., 2021		✓Hathorn et al., 2011	–
Pantothenic acid (B_5_)	–				✓Ismail et al., 2020; Patassini et al., 2019	
Pyridoxine (B_6_)	✓Aisen et al., 2008; De Jager et al., 2012; Seshadri et al., 2002; Smith, 2008; Kola et al., 2023	✓Murakami et al., 2010; Onaolapo and Onaolapo (2023); Kola et al., 2023	✓Seshadri et al., 2002; Smith, 2008	–	✓Sorolla et al., 2010	
Folic acid (B_9_)	✓Aisen et al., 2008; De Jager et al., 2012; Seshadri et al., 2002; Smith, 2008; Araújo et al., 2015; Clarke et al., 1998; da Silva et al., 2014; Murdaca et al., 2021; Quadri et al., 2004; Reynolds, 2006; Vogel et al., 2009; Kola et al., 2023	✓Murakami et al., 2010; Dos Santos et al., 2009; Xie et al., 2017; Obeid et al., 2007; Obeid et al., 2007; Kola et al., 2023	✓Seshadri et al., 2002; Smith, 2008; Araújo et al., 2015; Reynolds, 2006; Vogel et al., 2009	✓Obeid et al., 2007	–	–
Cobalamine (B_12_)	✓Aisen et al., 2008; De Jager et al., 2012; Seshadri et al., 2002; Smith, 2008; Araújo et al., 2015; Clarke et al., 1998; da Silva et al., 2014; Quadri et al., 2004; Vogel et al., 2009; Douaud et al., 2013; Lauer et al., 2022; McCaddon et al., 2002; McCaddon, 2013; O’Leary et al., 2012; Kola et al., 2023	✓McCaddon, 2013; Murakami et al., 2010; Obeid et al., 2007; Kola et al., 2023	✓Seshadri et al., 2002; Smith, 2008; Araújo et al., 2015; Vogel et al., 2009; McCaddon, 2013	✓McCaddon, 2013; Obeid et al., 2007	–	✓McCaddon, 2013
Vitamin A	✓da Silva et al., 2014; da Silva et al., 2014	✓da Silva et al., 2014	–	–	–	–
Vitamin C	✓da Silva et al., 2014; Murdaca et al., 2021; Arslan et al., 2020; Kola et al., 2023	✓Miyaue et al., 2022; Nagayama et al., 2004; Sudha et al., 2003; Kola et al., 2023	–	–	–	–
Vitamin D	✓da Silva et al., 2014; Annweiler et al., 2013; Balion et al., 2012; Chai et al., 2019; Dursun and Gezen-Ak, 2019; Kang et al., 2022; Littlejohns et al., 2014; Kola et al., 2023	✓Evatt et al., 2011; Luo et al., 2018; Lv et al., 2014; Pignolo et al., 2022; Rimmelzwaan et al., 2016; Kola et al., 2023	✓Chai et al., 2019; Littlejohns et al., 2014; Sommer et al., 2017	✓Bagur et al., 2017; Evans et al., 2018; James et al., 2013; Munger et al., 2006; Pierrot-Deseilligny, 2009; Smolders et al., 2019	–	✓De Marchi et al., 2023
Vitamin E	✓da Silva et al., 2014; Arslan et al., 2020; Icer et al., 2021; Kola et al., 2023	✓Icer et al., 2021; Kola et al., 2023	–	✓Salemi et al., 2010	✓Peyser et al., 1995	✓Icer et al., 2021
Copper	✓* da Silva et al., 2014; Cilliers, 2021; Fei et al., 2022; Moynier et al., 2020; Sasanian et al., 2020; Smith et al., 2007; Socha et al., 2021	–	–	–	–	–
Zinc	✓* da Silva et al., 2014; Cilliers, 2021; Fei et al., 2022; Moynier et al., 2020; Bush et al., 1994; Watt et al., 2011; Esler et al., 1996	–	–	–	–	–
Iron	✓* da Silva et al., 2014; Cilliers, 2021; Fei et al., 2022; Mantyh et al., 1993; Bulk et al., 2020; Madsen et al., 2020; Wang et al., 2019	✓Abeyawardhane and Lucas, 2019	–	–	–	–
Selenium, silicon, manganese, and arsenic	✓* Fei et al., 2022; Vicente-Zurdo et al., 2020	–	–	–	–	–
Manganese, calcium, and magnesium	✓Cilliers, 2021	–	–	–	–	–

*Increased levels, not a deficiency, of this micronutraceutical was linked to the associated neurodegenerative disease.

In migraine research, two particular micronutraceuticals have been investigated: riboflavin (Licina et al., 2023; Marashly and Bohlega, 2017; Nambiar et al., 2011; Plantone et al., 2021; Schoenen et al., 1998; Thompson and Saluja, 2017; Yamanaka et al., 2021) and magnesium (Domitrz and Cegielska, 2022; Licina et al., 2023; Maier et al., 2020). Various forms of epilepsy, which are receiving increasing amounts of research, respond to supplementation with vitamins B_6_ (Fox and Tullidge, 1946; Gospe, 2002; Kim and Cho, 2019), and B_9_ (Obeid et al., 2007), and D and E (Kim and Cho, 2019). Another well-researched neurological disorder is Wernicke-Korsakoff syndrome, which is linked specifically to vitamin B_1_ deficiency and alcoholism (Licina et al., 2023; Marashly and Bohlega, 2017; Nambiar et al., 2011; Plantone et al., 2021; Schoenen et al., 1998; Thompson and Saluja, 2017; Yamanaka et al., 2021; Isenberg-Grzeda et al., 2012; Krzysztoforska et al., 2023; Sechi and Serra, 2007). However, exploring all possible neurological disorders associated with micronutraceuticals is far too broad and beyond the scope of this review. Instead, Table 2 presents the major neurodegenerative diseases.

While minerals are not discussed in detail in this review, Table 2 shows that AD has been linked to the perturbation of several minerals. One mineral in particular—iron—has been observed to accumulate in the plagues of AD cases and to be neurotoxic; whereas, in Parkinson’s disease (PD) cases, iron is deficient (Table 2). As shown in Table 2, iron has not been directly linked to other major neurodegenerative conditions; however, it should be noted that iron is used in various mitochondrial enzyme components. Hence, a deficiency of iron (anemia) leads to perturbed neuroenergetics. Furthermore, various forms of anemia are often linked to a lack of bioavailability of zinc and vitamins A, B_6_, B_9_, B_12_, C, and E (Bhadra and Deb, 2020; Fishman et al., 2000). Hence, excessive or insufficient iron is associated with the pathogenesis and progression of neurodegenerative conditions.

Further inspection of Table 2 reveals that vitamins B_2_, B_3_, B_5_, and A are somewhat neglected in research on neurodegenerative diseases. In their 2014 review, Ashoori and Saedisomeolia (2014) identified vitamin B_2_ as a neglected micronutraceutical in neuronal research. Similarly, Miller and Dulay declared in their 2008 study that “the function of niacin in the brain has not yet been studied” (Miller and Dulay, 2008). However, since 2014, studies using a mouse model (Zhao et al., 2018) and a yeast model (Chen et al., 2020) have shown that vitamin B_2_ ameliorates AD. In 2023, Kodam et al. (2023) used an integrated multi-omics approach (transcriptomics, proteomics, and metabolomics) to identify deregulated neuroenergetics in patients with AD modulated by impaired metabolism involving vitamins B_2_, B_5_, and B6. In 2014 and 2015, studies on PD by the research team of Wakade, Chong, and Morgan reported the role of niacin in the management of symptoms of PD (Wakade and Chong, 2014; Wakade et al., 2015). Thus, the roles of the coenzymes NAD | NADH, FAD | FADH_2_, and FMN | FMNH_2_ in neurodegenerative diseases require more attention. In fact, recent research strategies have “gone back to basics” and are (re)examining the role of neuroenergetics in neurological disease. Two such AD studies, one by Xu et al. (2020) and another by Sang et al. (2022), took a closer look at the ubiquitous compound CoA and found a widespread deficiency in vitamin B_5_ in AD patients. A 2019 study on deficient neuroenergetics in HD also identified vitamin B_5_ deficiency (Patassini et al., 2019). Finally, in their in-depth and comprehensive systematic review and meta-analysis of AD, da Silva et al. (2014) found nine AD studies that measured vitamin A; however, the role of vitamin A in AD is still inconclusive, with only four studies reporting significantly decreased levels, whereas nine studies reported no changes. Hence, the role of these neglected vitamins (B_2_, B_3_, B_5_, and A) in neurodegenerative diseases should be a focal point for future research.

The most researched micronutraceuticals in neurodegenerative diseases are neurotropic B vitamins (B_6_ | B_9_ | B_12_), as well as vitamins D and E (see the studies listed in Table 2). Onaolapo and Onaolapo (2023) described the relationship between parkinsonism, vitamin B_6_, and the phosphorylated form of B_6_ (pyridoxal phosphate) as quite complex. As described in the previous section, all these vitamins play a significant role in maintaining good neuronal health; hence, it is no surprise that these vitamins are the focus of research on neurodegenerative diseases.

As a point of note, it must be noted that several studies have reported no correlation between vitamin supplementation and the alleviation of symptoms of neurodegenerative diseases (Kennedy, 2016). This begs the question: Why? Why are there conflicting outcomes regarding the efficacy of micronutraceuticals against neurodegenerative diseases and brain health in these studies? With this question in mind and the hot topics identified in this review, herein lies the motivation for the last section of this review: identifying the research gaps that exist in the literature and articulating the directives needed for future research in this scientific field.

## 5 Research gaps and directives for future research

Based on this review of the literature on the role of micronutraceuticals in neuronal health, it is evident that there are several gaps in the existing knowledge. These research gaps and suggestions for future studies are articulated here.

1) Many studies examining normal brain function have focused only on a small subset of the B vitamins (B_6_ | B_9_ | B_12_) (Kennedy, 2016). Other B vitamins and other micronutraceuticals are largely ignored. Instead of focusing on a small subset of micronutraceuticals in brain health, future research should investigate the potential effects of acute and chronic administration of a full range of micronutraceuticals and their interactions.
2) Studies that examine neurological diseases often neglect vitamins such as B_2_, B_3_, and B_5_. The role of these neglected micronutraceuticals in terms of the allostasis of neuroenergetics during the early onset, progression, and establishment of neurological diseases needs to be examined more closely.
3) The exact mechanisms of action of B vitamins in neurological diseases are unclear (Calderón-Ospina and Nava-Mesa, 2020). Future clinical studies that directly compare the effects of B vitamins in patients with neuropathology are needed to test their synergistic effects.
4) It is clinically difficult to isolate the effect of a single micronutraceutical on brain development because studies typically investigate the combined effects of multiple micronutrients (Tardy et al., 2020). Robust empirical data are needed to test the theoretical hypotheses regarding the effects of individual micronutraceuticals on brain development and function. Additionally, more validated experimental models and clinical data on (relatively ignored) younger and older populations are needed.
5) More studies are needed to identify biomarkers that can be used to assess the effects of micronutraceutical deficiencies (Sechi et al., 2016). New and improved methods are needed for the early detection of dysregulated/deficient micronutraceutical levels, before the onset of neurological symptoms, to prompt corrective treatment.
6) There are several conflicting studies on the therapeutic potential of micronutraceuticals in neurodegenerative diseases. More validation studies are needed to determine whether vitamin supplementation improves neurodegenerative diseases and to determine the optimal dosage and route of micronutraceutical administration.
7) Lack of application of micronutraceuticals laboratory findings in neurodegenerative diseases to clinical practice. Future clinical trials must be designed effectively and objectively to evaluate the progression, or lack thereof, of neurodegenerative diseases in patients receiving individual or combined vitamin supplementation.
8) There is a paucity of clinical studies on the role of micronutraceuticals in the pathogenesis and potential treatment of neurological disorders (Lahoda Brodska et al., 2023; Rai et al., 2021). Clinical studies are needed to elucidate the mechanism(s) by which micronutraceuticals affect the (anti)inflammatory and (anti)oxidative responses in the brain (i.e., evaluate the efficacy and protective role of micronutraceuticals in neurological disorders).
9) Explore the interaction between micronutraceuticals and environmental and lifestyle factors (such as exercise, stress, sleep, etc.) in neurological health to provide more comprehensive, personalized health recommendations.

With the abovementioned nine points in mind, research should be driven to garner mechanistic insights into the role of micronutraceuticals in neuronal health, validate their potential in ameliorating neurodegenerative diseases, and drive their application to personalized medicine (Badaeva et al., 2023).

## 6 Concluding remarks

This review highlights the role of micronutraceuticals in neuroenergetics, normal neuronal functioning, and health; and provides a summary of some of the neurological consequences of perturbed micronutraceuticals. The focus of this review was on neurons; however, neurons do not function independently but rely on support from glial cells (e.g., astrocytes and oligodendrocytes) to assist in neuronal function. Additional reviews are needed that cover the topic of neuronal support. It must also be stated that this review is by no means an exhaustive nor comprehensive overview of the literature. Specifically, one-carbon metabolism and the role of B vitamins in the homocysteine-methionine cycle and methylation, albeit important metabolic functions, were not discussed in this review. Other related topics not covered in this review include: various forms of brain cancer (gliomas), physical (traumatic) brain injury, neuroinfectious diseases (e.g., meningitis, encephalitis), psychiatric conditions, and cognitive decline, sensory disorders, and neurodiverse conditions (such as autism). Instead, the novelty offered by this review is the collective insights into existing research gaps and the provision of directives for future research.

We are at an inspiring stage in scientific research on the brain and its disorders. Various studies that have involved controlled interventions in large cohorts of participants have begun to demonstrate the mechanistic functions of micronutraceuticals in the brain. Based on the knowledge presented in this review, it is clear that the onset and/or severity of the increasing number of neurodegenerative diseases that occur in our society can be ameliorated by personalized medicine, whereby micronutraceuticals are actively monitored and adjusted accordingly via dietary supplementation. More research, driven by the research gaps and directives presented in this review, is needed to validate the roles of micronutraceuticals in neurodegenerative diseases.
